# Evidence for acquisition of virulence effectors in pathogenic chytrids

**DOI:** 10.1186/1471-2148-11-195

**Published:** 2011-07-08

**Authors:** Guiling Sun, Zefeng Yang, Tiffany Kosch, Kyle Summers, Jinling Huang

**Affiliations:** 1 Department of Biology, East Carolina University, Greenville, NC 27858, USA

## Abstract

**Background:**

The decline in amphibian populations across the world is frequently linked to the infection of the chytrid fungus *Batrachochytrium dendrobatidis *(*Bd*). This is particularly perplexing because *Bd *was only recently discovered in 1999 and no chytrid fungus had previously been identified as a vertebrate pathogen.

**Results:**

In this study, we show that two large families of known virulence effector genes, crinkler (CRN) proteins and serine peptidases, were acquired by *Bd *from oomycete pathogens and bacteria, respectively. These two families have been duplicated after their acquisition by *Bd*. Additional selection analyses indicate that both families evolved under strong positive selection, suggesting that they are involved in the adaptation of *Bd *to its hosts.

**Conclusions:**

We propose that the acquisition of virulence effectors, in combination with habitat disruption and climate change, may have driven the *Bd *epidemics and the decline in amphibian populations. This finding provides a starting point for biochemical investigations of chytridiomycosis.

## Background

A recent report by IUCN indicates that amphibians are the most endangered group of vertebrates worldwide, with 32 percent listed as threatened with extinction and 159 species already believed to be extinct [1]. Among the several factors often linked to the decline in amphibian populations is the chytrid fungus *Batrachochytrium dendrobatidis *(*Bd*), the causative agent of chytridiomycosis. Ever since the discovery of *Bd *in 1999 [2], numerous cases have been reported for the destruction associated with chytridiomycosis [3,4]. The destructive path of chytridiomycosis fits all of the descriptors of a novel epidemic (i.e. sudden appearance, strong virulence, and rapid transmission) [5]. Investigations of museum specimens collected from as far back as 1902 in Japan [6] and 1938 in Africa [7] have detected *Bd *in skin cross sections. On the other hand, long-term population studies since the 1900's had no records of mass mortalities until the late 1970's when the first *Bd *epidemics were reported [8]. This leads to an interesting question about how pathogenesis suddenly evolved in this previously benign organism.

Many disease organisms existed in non-virulent/commensal forms until they acquired some novel traits allowing them to exploit new niches [9]. Among the several possible underlying mechanisms, horizontal gene transfer (HGT) is the most rapid way for an organism to gain novel phenotypes [10] and it has contributed to the origin and spread of pathogenesis in numerous bacteria [9,11] and occasionally in eukaryotes [12,13]. In this study, we show that homologs of two large families of known virulence effector genes were likely acquired by *Bd*. These families have been rapidly duplicated after their acquisition in *Bd *and evolved under strong positive selection. We hypothesize that the acquisition of these virulence effector homologs may partly explain the evolution of chytridiomycosis.

## Results and Discussion

Phylogenomic analyses identified homologs of two large families of known virulence effectors in *Bd*, including serine peptidases and CRN proteins (Table 1; Additional file 1). Of these, members of the serine peptidase family have been implicated as virulence effectors in *Bd *because of their potential role in degrading host antimicrobial peptides [14]. Previous microarray data also indicated that many serine peptidase genes in *Bd *are highly expressed in the sporangia stage, which is associated with the infection of keratinized host tissue [14]. Our analyses show that at least 32 serine peptidase genes are present in the *Bd *genome (Additional file 1). Other identifiable homologs of *Bd *serine peptidases are only found in bacteria, where they are more broadly distributed.

**Table 1:** Structures and predicted subcellular localization of the CRN protein family in *Bd *JAM 81.

JGI IDs	Domain structure		Introns	Scaffold location/gene number in the scaffafold	ESTs	TargetP	SignalP		iPSORT
							
	N-	C-					NN	HMM	
26417	typeA	DXX DHA	1	scaffold_10/377		_		_	_
26603	typeA	DXX DHA	2	scaffold_10/377		_	_	_	_
26694		DN17	0	scaffold_10/377		_	_	_	_
90730	typeA	DXX DHA	1	scaffold_10/377		_	_	_	_
90240	typeA	DXX DHA	1	scaffold_9/369		_	_	_	_
26137	typeA	DXX DHA	6	scaffold_9/369		_	_	_	_
26152	typeA	DXX DHA	3	scaffold_9/369		_	_	_	_
26186	typeA	DXX DHA	0	scaffold_9/369		_	_	_	_
90292		DN17	1	scaffold_9/369		_	_	_	_
90343	typeB	DXX DXV	0	scaffold_9/369	6	_	_	_	_
26410	typeA	DXX DHA	5	scaffold_9/369		_	_	_	_
23764	typeA	DXX DHA	2	scaffold_4/596	1	_	_	_	_
34850	typeB	DFB	0	scaffold_4/596	10	_	_	_	_
87524	typeB	DFB	0	scaffold_4/596	12	_	_	_	_
24811	typeA	DXX DHA	2	scaffold_6/516		_	_	_	_
88508	typeA	DXX DHA	0	scaffold_6/516	2	_	_	_	_
25057	typeB	DX8	0	scaffold_6/516	3	_	_	_	_
84882	typeA	DXX DHA	0	scaffold_1/1593	1	_	_	_	_
84908	typeA	DXX DHA	0	scaffold_1/1593		_	_	_	_
34275	typeA	DXX DHA	3	scaffold_1/1593	2	_	_	_	_
22414	typeA	DXX DAB	1	scaffold_1/1593		_	_	_	_
85109	typeA	DXX DHA	0	scaffold_1/1593	1	_	_	_	_
22610	typeA	DXX DHA	6	scaffold_1/1593	1	_	_	_	_
22615	typeA	DXX DHA	1	scaffold_2/629		_	_	_	_
86546	typeB	DFA DDC	0	scaffold_2/629	1	_	_	_	_
86517		DN17	1	scaffold_2/629		_	_	_	_
36642		DBE	0	scaffold_2/629	2	_	S	S	_
23071	typeB	DX8	4	scaffold_2/629	4	_	_	_	_
23077	typeB	DX8	3	scaffold_2/629	3	_	_	_	_
23173	typeA	DXX DAB	3	scaffold_2/629	1	_	_	_	_
23206	typeA	DXX DHA	4	scaffold_3/601		_	_	_	_
23217		DN17	1	scaffold_3/601		_	_	_	_
87221	typeB	DX8	0	scaffold_3/601	2	_	S	_	_
87128		DN17	0	scaffold_3/601		_	_	_	_
23760	typeA	DXX DHA	1	scaffold_3/601	1	_	_	_	_
25252	typeA	DXX DHA	5	scaffold_7/438		_	_	_	_
25352	typeB	DXX DXV	0	scaffold_7/438	2	_	_	_	_
35207		DX8	1	scaffold_7/438	3	_	_	_	_
37012	typeB	DFB	0	scaffold_7/438	10	_	_	_	_
25664	typeA	DXX DXV	1	scaffold_7/438		_	_	_	_
91252	typeA	DXX DHA	1	scaffold_12/268		_	_	_	_
91239		DFB	1	scaffold_12/268	8	_	_	_	_
91238	typeA	DXX DHA	1	scaffold_12/268	1	_	_	_	_
27205	typeA	DXX DHA	2	scaffold_12/268		_	_	_	_
35892	typeB	DFA DDC	0	scaffold_12/268	6	_	_	_	_
91331	typeA	DXX DHA	1	scaffold_12/268		_	_	_	_
89833		DFB	0	scaffold_8/465	8	_	_	_	_
89826	typeA	DXX DHA	1	scaffold_8/465		_	_	_	_
24299	typeA	DXX DHA	3	scaffold_5/527		_	_	_	_
24333	typeB	DFB	0	scaffold_5/527	14	_	_	_	_
24341	typeA	DXX DAB	2	scaffold_5/527		_	_	_	_
36889	typeB	DFB	0	scaffold_5/527	12	_	_	_	_
87953	typeB	DFB	0	scaffold_5/527	13	_	S	_	_
87954	typeB	DFB	0	scaffold_5/527	14	_	S	_	_
88475	typeA	DXX DHA	0	scaffold_5/527		_	_	_	_
24764	typeB	DFA DDC	0	scaffold_5/527	2	_	_	_	_
88482	typeA	DXX DHA	2	scaffold_5/527	1	_	_	_	_
27291	typeA	DXX DHA	3	scaffold_13/279		_	_	_	_
37407	typeB	DFB	0	scaffold_13/279	14	_	_	_	_
27710	typeA	DXX DHA	1	scaffold_14/258	1	_	_	_	_
27719	typeA	DXX DHA	0	scaffold_14/258		_	_	_	_
36061	typeB	DX8	0	scaffold_14/258	6	_	_	_	_
28349	typeA	DXX DXV	1	scaffold_18/194		_	_	_	_
28355	typeA	DXX DHA	0	scaffold_18/194		M	_	_	_
92602	typeA	DXX DHA	0	scaffold_18/194		_	_	_	_
28463	typeA	DXX DHA	4	scaffold_18/194		_	_	_	_
28466	typeA	DXX DXV	1	scaffold_18/194	1	_	_	_	_
92692	typeB	DFA DDC	1	scaffold_18/194	3	_	_	_	_
28183	typeA	DXX DHA	4	scaffold_17/196		_	_	_	_
28329	typeA	DXX DHA	3	scaffold_17/196		_	_	_	_
28345	typeA	DXX DHA	1	scaffold_17/196		_	_	_	_
28523		DXX DHA	2	scaffold_19/95		S	S	_	_
28594		DN17	3	scaffold_19/95	1	_	_	_	_
28683	typeA	DXX DHA	3	scaffold_20/92		_	_	_	_
28687	typeA	DXX DHA	3	scaffold_20/92		_	_	_	_
26749	typeA	DXX DHA	4	scaffold_11/338		S	S	_	_
90966		DX8	0	scaffold_11/338	1	_	S	_	_
26764	typeA	DXX DAB	3	scaffold_11/338		_	_	_	_
26962	typeB	DX8	0	scaffold_11/338	7	_	S	_	_
26980	typeB	DX8	0	scaffold_11/338	6	_	S	_	_
91063	typeA	DXX DHA	1	scaffold_11/338	1	_	_	_	_
92027	typeA	DXX DHA	1	scaffold_15/227		_	_	_	_
27784	typeA	DXX DAB	2	scaffold_15/227		_	_	_	_
92094	typeA	DXX DHA	1	scaffold_15/227		_	_	_	_

"Type A" and "Type B" show two different types of conserved N-terminal domains of *Bd *CRN proteins, whose detailed information is shown in Additional file 5. "M" and "S" show that the protein is likely localized in mitochondria and the secretory pathway, respectively.

The CRN protein family consists of cytoplasmic virulence effectors only known in oomycete plant pathogens [15,16]. These effectors are referred to as "crinkler" proteins because of their association with cell death and leaf crinkling, which parallels the effects of *Bd *on amphibian skin [17]. In oomycete pathogens, the N-terminal region of CRN proteins contains a highly conserved LFLAK domain that is characteristic of all CRN proteins. The C-terminal region is diverse in domain and motif structure, based on which 24 subfamilies were identified in the oomycete *Phytophthora infestans *[16]. The C-terminal region of CRN proteins also controls virulence and, when expressed in plant cells, may induce cell death [16]. Our analyses identified 84 genes encoding CRN protein homologs in *Bd *(Table 1). These genes are present in genomes of both *Bd *isolates (JAM81 and JEL423) and include eight of the 24 subfamilies of CRN proteins known in oomycetes (Table 2).

**Table 2:** Comparison of gene copy numbers of CRN protein subfamilies in *Bd *and oomycetes

Subfamilies	*Bd*-JAM81	*Bd*-JEL423	*Pi*	*Ps*	*Pc*	*Pr*	*Pu*	*Pb^1^*	*Pp^1^*	*Ae^1^*
DBE^2^	1	1	1	2	2				y	
DFA-DDC	4	5	1	2	2				y	y
DFB	10	11	8	2	3		1	y		
DXX-DAB	5	5	8	3						
DXX-DHA^2^	44	30	9	1	1					
DXX-DXV^2^	5	5	7	3	1					
DN17	6	6	10	10	4	2		y		y
DX8	9	7	7	4				y		

Abbreviations: *Pi*, *Phytophthora infestans*; *Ps*, *Phytophthora sojae*; *Pc*, *Phytophthora capsici*; *Pr*, *Phytophthora ramorum*; *Pu*, *Pythium ultimum*; *Pb*, *Phytophthora brassicae*; *Pp*, *Phytophthora parasitica*; *Ae*, *Aphanomyces euteiches*.

^1^Sequences were found in EST data.

^2^Homologs are present in green plants. All other subfamilies are solely restricted in *Bd *and oomycetes.

Compared to oomycetes, the number of genes in each subfamily is often higher in *Bd*. In particular, the DXX-DHA subfamily of CRN proteins is highly duplicated in *Bd *and contains 30 and 44 gene copies from the two respective genomes, many of which show very little sequence variation. The homology of CRN proteins from *Bd *and oomycetes is most obvious in the C-terminal effector region, with a sequence identity of up to 46.5%. The N-terminal LFLAK domain characteristic of oomycete CRN proteins also is largely conserved in some identified CRN homologs in *Bd *(Additional file 2). As in oomycete pathogens [16,18], only a fraction of CRN homologs identified in *Bd *are predicted to contain signal peptides (Table 1). However, almost all other identified CRN homologs in *Bd *have short N-terminal extensions of about 20 amino acids in positions corresponding to signal peptides (Table 1 and Additional file 2).

**Figure 1:**
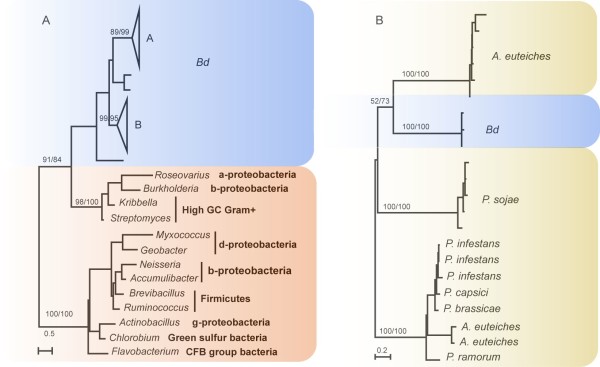
**Molecular phylogenies of serine peptidases (A) and the DN17 subfamily of CRN proteins (B)**. Numbers above major branches show bootstrap values from maximum likelihood and distance analyses, respectively. The DN17 tree is rooted at the two distinct sequence copies (groups) in oomycetes. Genes from *Bd*, oomycetes and bacteria are indicated in different colors. *P *= *Phytophthora*; *A *= *Aphanomyces*.

The observation that a gene is uniquely shared by distantly related organisms can be attributed to multiple possible scenarios, in particular differential loss and HGT [19,20]. Although differential loss and HGT can always be invoked as alternative explanations to each other, each scenario becomes increasingly less likely if it requires considerably more corresponding (gene loss or HGT) events [19,21]. In the cases of serine peptidases and CRN proteins, because chytrids and oomycetes belong to opisthokonts and heterokonts respectively, the gene loss scenario entails numerous independent loss events in related taxa. A more parsimonious and plausible scenario is the occurrence of HGT between *Bd *and oomycetes. To further investigate the number and the direction of potential HGT events involved, we performed phylogenetic analyses on serine peptidases and each of the eight CRN subfamilies shared between *Bd *and oomycetes. Our analyses indicate that all serine peptidase homologs in *Bd *form a weakly supported clade (Figure 1A). Given that serine peptidases are more widely distributed in bacteria, it is most likely that serine peptidases in *Bd *are derived from a single HGT event from bacteria, followed by gene duplication. Analyses of CRN subfamilies show that, in most cases, sequences from *Bd *form a distinct clade whereas those from oomycetes form another, suggesting independent HGT for each CRN subfamily. With paralogous sequence clades as outgroup, our analyses of the DN17 subfamily also show that *Bd *sequences were likely acquired from oomycetes, though the support is still modest (Figure 1B). Such low support is expected given the short length of sequences used in our phylogenetic analyses. Additional evidence consistent with the direction of HGT from oomycetes comes from their higher CRN gene diversity. The fewer CRN subfamilies in *Bd *(8 versus 24 in oomycetes) are in line with the transfer from oomycetes, as we would expect the recipient organism to receive a subset of the gene repertoire of the donor. Nevertheless, for both serine peptidases and the CRN family, we were unable to determine the timing of these HGT events because of the paucity of sequence data from other chytrids and oomycetes.

Because both the serine peptidase and CRN families are highly duplicated in *Bd*, we reasoned that they may confer important functions and, therefore, investigated the possibility that strong selection has been involved in their evolution. The estimate of ***d_N_*/*d_S _***for the serine peptidase family in *Bd *is 0.46, while this value is 0.01 in bacteria. Similarly, ***d_N_*/*d_S _***for each of seven CRN subfamilies (with more than three sequences) is significantly higher in *Bd *than in oomycetes (Table 3). These results indicate that both the serine peptidase and CRN families evolved faster in *Bd *than in oomycetes and bacteria. We further used three likelihood tests (M2a *vs*. M1a, M8 *vs*. M7 and M8 *vs*. M8a) [22] to identify the role of positive selection on the serine peptidase family and seven CRN subfamilies in *Bd *and in oomycetes or bacteria, respectively. *A priori *we stipulated that evidence for positive selection will be sufficient only if all three tests performed show statistical significance. In total, all eight families and subfamilies in *Bd *show strong evidence of positive selection (Table 3 ; Additional file 3). In contrast, we found that only four subfamilies of CRN proteins (DX8, DXX-DXV, DXX-DHA, and DN17) evolved under positive selection in oomycetes. For the subfamilies that were influenced by positive selection in both *Bd *and oomycetes, the improved branch-site model [23] was used to identify positive selection specifically on the diverging *Bd *lineages. The genes in *Bd *were used as foreground branches and those in oomycetes were used as background. Except for the subfamily DXX-DHA, likelihood tests for the other three CRN subfamilies show statistically significant support for positive selection in the *Bd *branch (Additional file 3). Furthermore, multiple positively selected amino acid sites were identified using the Bayes Empirical Bayes (BEB) estimation procedure [24], suggesting that positive selection acted specifically on certain amino acid sites of the serine peptidase family and the CRN subfamilies after their acquisition by *Bd*.

**Table 3:** Summary of evolutionary rate and positive selection analyses

Gene family	Lineage	*d*_*N*_/*d*_*S*_^1^	Positive selection^2^	PSS^3^
Serine peptidase	*Bd*	0.46	+	30, 19*, 14**
	bacteria	0.01	-	-

DXX-DAB	*Bd*	0.70	+	43, 8*
	oomycetes	0.40	-	-

DFB	*Bd*	0.64	+	90, 21*, 11**
	oomycetes	0.27	-	-

DX8	*Bd*	0.88	+	101, 18*, 8**
	oomycetes	0.52	+	41, 12*, 5**

DXX-DXV	*Bd*	0.65	+	114, 2*
	oomycetes	0.59	+	27, 16*, 12**

DXX-DHA	*Bd*	0.78	+	61, 18*, 8**
	oomycetes	0.63	+	28, 11*, 4**

DFA-DDC	*Bd*	0.69	+	36, 17*, 8**
	oomycetes	0.26	-	-

DN17	*Bd*	0.50	+	25, 8*, 3**
	oomycetes	0.47	+	29, 13*, 9**

^1^The *d_N _*/*d_S _*ratio average across all sites and lineages under PAML model M0.

^2^"+" signs indicate that positive selection acted on the evolution of these genes, while "-" signs indicate that no positive selection was identified. These results were obtained based on the likelihood ratio tests of three site-specific models (Additional file 1).

^3^PPS indicates positively selected sites (*ω *> 1 under PAML model M2a). The number of amino acid residues with the posterior probability (PP) of positive selection greater than 0.05 is shown. **: PP > 0.99; *: PP > 0.95.

Most genes of the serine peptidase family in *Bd *were assigned to two clades in the phylogeny with strong bootstrap support (Figure 1A). We estimated the coefficients of both type I (a site-specific shift in evolutionary rate) [25] and type II functional divergence (a cluster-specific shift of amino acid property) [26] between them. The results showed that only the type I coefficient of functional divergence is significantly greater than zero (*θ *_I _= 0.5744 ± 0.0589, LRT = 220.3270, *p *< 0.01). These results suggest that site-specific selective constraints have been altered for most of the members of this family, leading to group-specific functional evolution after their diversification. Furthermore, 26 amino acid residues were found to be critical for type I functional divergence with a posterior probability higher than 0.95 (Additional file 3), indicating that the evolutionary rates for these sites have shifted between these two clades. Some amino acid residues in the region of the Peptidase_S41 domain may also have contributed to the functional divergence, as there are 11 critical residues that are located in this region.

The acquisition of the serine peptidase and CRN families followed by rapid duplication and positive selection suggests that these acquired genes are likely important in the adaptation of *Bd*. Because both families are known virulence effectors, their presence in *Bd *provides a starting point for biochemical investigations of chytrid pathogenesis. If members of these two families are indeed related to *Bd *virulence, their acquisition may at least partly explain the emergence of chytridiomycosis. Peptidases are ubiquitous tools of bacterial parasites that enable host infection, and have also been identified as key factors in fungal pathogenesis. For example, *Microsporum canis*, a dermatophytic fungal parasite, infects keratinized tissue (e.g. skin and nails) in mammals. This fungus secretes dipeptidyl peptidases, which are homologous to serine peptidases of the S9 family in bacteria [27]. These and other proteases enable the fungus to obtain nutrients from the complex network of insoluble proteins that comprise keratin-rich tissues. As mentioned above, serine peptidases were identified as a class of proteins that are associated with virulence in *Bd *using microarray expression analyses [14]. The CRN genes were previously only known in oomycetes, which themselves also are important pathogens. Although most attention has been paid to oomycete plant pathogens, there is also a diverse group of oomycetes that infect animals [28]. Some of these pathogens are responsible for epidemics in aquatic animals such as crayfish [28]. The oomycete parasite *Saproglenia *may induce high mortality in salmon populations, both in aquaculture and in the wild [29], with effects similar to *Bd *[30]. Hence, the oomycete lineage is well adapted to parasitize vertebrate hosts. It is not surprising that oomycetes have an arsenal of genes associated with parasitism and that the acquisition of such genes may help convert a commensal chytrid to a parasitic pathogen.

## Conclusions

Our data show that two large families of known virulence effector genes, CRN proteins and serine peptidases, were likely transferred to *Bd *from oomycete pathogens and bacteria, respectively. These acquired virulence effector homologs have been under rapid gene duplication and strong positive selection, suggesting that they may play an important role in the adaptation of *Bd*. Given that pathogenesis is the outcome of microbe-host interactions, the impact of any virulence effector is likely more severe when the host becomes stressed. We hypothesize that the acquisition of virulence effectors, in combination with habitat disruption and climate change, led to the *Bd *epidemics and the decline in amphibian populations. Further investigations are warranted to fully understand the biological functions of virulence effector homologs identified in our analyses.

## Methods

### Data source and genome screening for HGT candidates

Phylogenomic analyses used a customized database combining sequences from the NCBI non-redundant (*nr*) database, dbEST, the Taxonomically Broad EST Database (TBestDB) [31] and additional eukaryotic genomes. Annotated protein sequences of the heterokont *Aureococcus anophagefferens*, haptophyte *Emiliania huxleyi*, green algae *Chlorella sp*. and *C. vulgaris*, metazoans *Daphnia pulex*, *Capitella sp*. and *Lottia gigantea *were obtained from the Joint Genome Institute http://www.jgi.doe.gov. The annotated genome of the red alga *Cyanidioschyzon merolae *was downloaded from the *Cyanidioschyzon merolae *Genome Project http://merolae.biol.s.u-tokyo.ac.jp, and those of fungi *Rhizopus oryzae*, *Allomyces macrogynus*, and *Spizellomyces punctatus *were obtained from their sequencing projects at the Broad Institute of Harvard and MIT http://www.broadinstitute.org. Detailed sources for annotated protein sequences and ESTs of two *Bd *isolates and seven oomycetes used in our analyses are shown in Additional file 4. A comprehensive database was created using above data.

Genome screening for horizontally acquired genes was performed using AlienG [32]. AlienG is based on the observation that sequence similarity is correlated to sequence relatedness, where sequence similarity is measured by BLAST bit scores. A query sequence is considered to be a candidate of HGT-derived gene if it is significantly more similar to homologs from a potential donor (*S_d_*) than to those from closely related taxa (*S_r_*). Specifically, a bit score ratio *S_d_/S_r _*= 1.5 was used as threshold to identify candidates of HGT-derived genes in *Bd*. To eliminate potential artifacts, only candidates with expression evidence from ESTs or located in contigs containing more than 5 genes are retained for further analyses.

### Identification of serine peptidase and CRN protein families in Bd and oomycetes

Phylogenomic analyses identified homologs of serine peptidases and CRN proteins among the HGT candidates. To retrieve other CRN and serine peptidase homologs in *Bd*, we first downloaded bacterial serine peptidases and annotated oomycete CRNs from both GenBank and oomycete genome projects [16,18]; these downloaded sequences were then used as queries to search (BLASTP) against *Bd *JAM81 annotated protein sequences. All hits with E-values lower than 1 × 10^-5 ^were retained. To classify the retrieved CRN homologs in *Bd*, we followed the criteria used for the oomycete *P. infestans *[16], which is based on the structures of C-terminal specific domains. As a result, eight subfamilies of CRN homologs were identified in *Bd*. To further estimate the copy numbers for homologs of serine peptidases and each CRN subfamily in *Bd*, we first constructed profiles for the *Bd *serine peptidase domain and the C-terminal specific domain regions of each CRN subfamily using HMMER [33]. The generated profiles were used to search the *Bd *genome, and sequences with E-values lower than 1 × 10^-5 ^were retrieved and further aligned using conserved domain regions as references. Extremely divergent or partial sequences were removed from further analyses. The remaining genes were considered to be putative serine peptidases or CRN proteins. To exclude the possibility of contamination in *Bd *JAM81, the same process was also used to identify genes in the isolate *Bd *JEL423. Because the CRN protein family from four oomycetes (*P. infestans*, *P. sojae*, *P. ramorum*, and *Pythium ultimum*) have been well annotated [16,18], we also used their C-terminal specific domain regions as references to identify CRN proteins in other oomycete datasets, including the *P. capsici *genome and ESTs for three other oomycetes (*P. brassicae*, *P. parasitica*, and *Aphanomyces euteiches*). Partial sequences were excluded and the remaining genes were regarded as CRN proteins. Secretion signals of serine peptidases and CRN proteins were identified using three tools (SignalP [34], TargetP [35], and iPSORT [36]) with default settings. The conserved N-terminal profiles were generated using WebLogo http://weblogo.berkeley.edu.

### Phylogenetic analyses

Protein sequence alignment was carried out using MUSCLE [37] and clustalX [38], followed by visual inspection and manual refinement. Gaps and ambiguously aligned sites were removed manually. The most appropriate protein substitution matrix, rate heterogeneity and invariant sites were determined using ModelGenerator [39] for serine peptidases and each of the eight CRN subfamilies identified in *Bd*. Phylogenetic analyses were performed with a maximum likelihood method using PHYML [40] and a distance method using *neighbor *of PHYLIPNEW v.3.68 [41] in EMBOSS package [42]. Bootstrap support values were estimated using 100 pseudo-replicates. For distance analyses, maximum likelihood distances were calculated using TREE-PUZZLE v.5.2 [43] and PUZZLEBOOT v.1.03 (A. Roger and M. Holder, http://www.tree-puzzle.de. The models used in TREE-PUZZLE/BUZZLEBOOT to calculate the maximum likelihood distances were the same as those determined by ModelGenerator. All other parameters in the analyses used default settings.

### Detection of positive selection

The ***d_N_***/***d_S _***values for the serine peptidase family and seven CRN subfamilies (with more than three members in *Bd*) were estimated both in *Bd *and in oomycetes or bacteria, respectively. Genes with partial sequences were removed from the analyses. The program PAL2NAL [44] was used to convert protein sequence alignments into the corresponding codon-based DNA alignments. The program CODEML [22] was then used to calculate the ***d_N_***/***d_S _***values.

Maximum likelihood analyses of selective constraints on the serine peptidase family and seven CRN subfamilies were performed using PAML [22] with their phylogenies and the codon alignments as input. For the serine peptidase family and each CRN subfamily, we firstly used the site-specific models [22,45,46] to identify positively selected codon sites in *Bd *and in oomycetes or bacteria, respectively. In this analysis, we compared models M1a *vs*. M2a, M7 *vs*. M8 and M8a *vs*. M8 to identify positive selection. To assess whether positive selection acted on the evolution of each group, the log likelihood values for each pair of nested models were compared using a likelihood ratio test (LRT). In the LRT, twice the log likelihood difference (2Δ/) between the two models was compared with the *χ *^2 ^statistics, with degrees of freedom (DF) equal to the difference of the number of parameters. The DFs were 3, 2, and 1 for comparisons M1a *vs*. M2a, M7 *vs*. M8 and M8a *vs*. M8, respectively. If the LRT was statistically significant and ***d_N_***/***d_S _***estimate greater than 1, evidence for positive selection would be considered sufficient for the genes analyzed. Then, the Bayes Empirical Bayes method [24] was used to determine the amino acids most likely responsible for positive selection.

The improved branch-site models [23] were also used to search for positive selection for the serine peptidase family and each CRN subfamily. In these tests, we compared the null model A (model = 2, NSsites = 2, with ω fixed to 1) with the alternative model (model = 2, NSsites = 2). For each family or subfamily, the *Bd *branch on the phylogeny was used as foreground, while the oomycetes or bacteria branch used as background. When the LRT suggests positive selection under this model, the codon sites likely evolved under positive selection can be identified through posterior probability from Bayes Empirical Bays method [24].

### Analysis of functional divergence

The program DIVERGE [47] was used to estimate the coefficients of both type I (*θ _I_*) and type II (*θ _II_*) functional divergence between serine peptidase clades A and B. Because subfamilies with fewer than four sequences cannot be analyzed using this method, three other serine peptidase proteins were excluded from this analysis. A likelihood ratio test was performed to test the null hypothesis *θ = *0 using *χ *^2 ^distribution with 1 DF. A significant *θ _I _*indicates that site-specific altered selection constraints after the divergence of clades, while a significant *θ _II _*means site-specific shift of amino acid physiochemical property [25,26].

## Competing interests

The authors declare that they have no competing interests.

## Authors' contributions

JH designed the study and wrote the manuscript. GS and ZY performed the analyses and participated in manuscript writing. SK and KS wrote the manuscript and contributed to data analyses. All authors read and approved the final manuscript.

## Supplementary Material

Additional file 1 **Gene structures and subcellular localizations of serine peptidases in *Bd***. This file contains information about gene identifiers, numbers of exon, and predicted subcellular localizations for serine peptidases that were identified in *Bd *JAM 81. Click here for file

Additional file 2 **LFLAK domain of the CRN protein family in oomycetes and *Bd***. This file shows that the LFLAX domain of the CRN protein family is largely conserved between oomycetes and *Bd*. Click here for file

Additional file 3 **Results of selection analyses**. This file contains results of positive selection analyses using site-specific and branch-site models, respectively. It also shows amino acid residues that are critical to functional divergence between serine peptidase clades A and B. Click here for file

Additional file 4 **Data sources for *Bd *isolates and oomycetes used in the analyses**. This file shows data types and download sites for two *Bd *isolates and seven oomycetes used in the analyses. Click here for file

Additional file 5 **Two different conserved N-terminal domains of *Bd *CRN proteins**. This file shows two different types of N-terminal domain for *Bd *CRN proteins. These domain types are also indicated in Table 1. Click here for file
