# Supplementary material for: Evidence for acquisition of virulence effectors in pathogenic chytrids
Source: BMC Evol Biol. 2011 Jul 8;11:195. doi: 10.1186/1471-2148-11-195 (PMC3161006; doi:10.1186/1471-2148-11-195)
Supplement: Additional file 1 — Gene structures and subcellular localizations of serine peptidases in Bd. This file contains information about gene identifiers, numbers of exon, and predicted subcellular localizations for serine peptidases that were identified in Bd JAM 81. [file 1471-2148-11-195-S1.PDF]

Table S1. Domain structure, gene structure, and cellular localization prediction of serine peptidase family in *Batrachochytrium dendrobatidis* JAM81

JGI IDs	Exon numbers	Scaffold location/gene number in the scaffafold	EST numbers	TargetP	SignalP		iPSORT	Short N-terminus
					NN	HMM		
26098	2	scaffold_9*369		y	y	y	y	
26287	2	scaffold_9*369		y	y	y	y	
90146	2	scaffold_9*369		y	y	y	_	
24156	2	scaffold_4*596		y	y	y	y	
24207	1	scaffold_4*596		_	_	_	_	y
87928	1	scaffold_4*596		_	y	_	_	y
24985	2	scaffold_6*516		y	y	y	y	
25222	3	scaffold_6*516		y	y	y	y	
85649	2	scaffold_1*1593		y	y	y	y	
22176	2	scaffold_1*1593		y	y	y	y	
86001	2	scaffold_1*1593		y	y	y	y	
86231	2	scaffold_2*629		y	y	y	y	
86314	2	scaffold_2*629		y	y	y	y	
23310	2	scaffold_3*601		y	y	y	y	
23534	3	scaffold_3*601		_	_	_	_	y
23544	3	scaffold_3*601		y	y	_	_	
23754	2	scaffold_3*601		y	y	y	y	
25462	2	scaffold_7*438		y	y	y	y	
25463	2	scaffold_7*438		y	y	y	y	
25559	6	scaffold_7*438		y	y	_	_	
89445	1	scaffold_7*438		_	y	_	_	y
27246	2	scaffold_12*268		y	y	y	y	
35365	2	scaffold_8*465	1	y	y	y	y	
92476	1	scaffold_17*196		_	_	_	_	y
92744	2	scaffold_19*95		y	y	y	_	
28623	2	scaffold_20*92		y	y	y	y	
92841	1	scaffold_20*92		_	_	_	_	y
28625	1	scaffold_20*92		_	_	_	_	y
37569	2	scaffold_20*92	3	y	y	y	y	
27937	2	scaffold_15*227		y	y	y	y	
28775	2	scaffold_35*7		y	y	y	y	
93324	1	scaffold_212*2		_	_	_	_	y
