# Supplementary material for: Evidence for acquisition of virulence effectors in pathogenic chytrids
Source: BMC Evol Biol. 2011 Jul 8;11:195. doi: 10.1186/1471-2148-11-195 (PMC3161006; doi:10.1186/1471-2148-11-195)
Supplement: Additional file 3 — Results of selection analyses. This file contains results of positive selection analyses using site-specific and branch-site models, respectively. It also shows amino acid residues that are critical to functional divergence between serine peptidase clades A and B. [file 1471-2148-11-195-S3.DOC]

**Table S2. Results of positive selection using** site-specific models

| Gene family | Lineage | $d_{N}/d_{S}$ under M0^1^ | $2\Delta l$ M2a *vs.* M1^2^ | $2\Delta l$ M8 *vs.* M7^2^ | $2\Delta l$ M8 *vs.* M8a^2^ | M2a estimates | PSS under M2a^3^ | M8 estimates | PSS under M8^3^ |
| --- | --- | --- | --- | --- | --- | --- | --- | --- | --- |
| serine peptidase | *Bd* | 0.4585 | 185.34^**^ | 193.05^**^ | 148.36^**^ | $p_{0}=0.4931,\omega_{0}=0.1595$ $p_{1}=0.4739,\omega_{1}=1.0000$ $p_{2}=0.0690,\omega_{2}=3.4028$ | 30, 19^*^, 14^**^ | $p_{1}=0.0862,\omega=2.6007$ $\beta(p=0.6592,q=0.9363)$ | 37, 21^*^, 14^**^ |
|  | bacteria | 0.0118 | 0 | 0 | 2.39 | - | - | - | - |
| DXX-DAB | *Bd* | 0.6953 | 28.92^**^ | 28.86^**^ | 28.85^**^ | $p_{0}=0.8499,\omega_{0}=0.1103$ $p_{1}=0.0000,\omega_{1}=1.0000$ $p_{2}=0.1501,\omega_{2}=6.1007$ | 43, 8^*^ | $p_{1}=0.1408,\omega=6.3279$ $\beta(p=0.0535,q=0.3036)$ | 59, 13^*^, 6^**^ |
|  | oomycetes | 0.3929 | 0 | 0 | 0 | - | - | - | - |
| DFB | *Bd* | 0.6429 | 132.04^**^ | 131.28^**^ | 127.86^**^ | $p_{0}=0.6276,\omega_{0}=0.0000$ $p_{1}=0.2532,\omega_{1}=1.0000$ $p_{2}=0.1192,\omega_{2}=4.6036$ | 90, 21^*^, 11^**^ | $p_{1}=0.1676,\omega=3.8022$ $\beta(p=0.0050,q=0.0164)$ | 139, 59^*^, 24^**^ |
|  | oomycetes | 0.2704 | 0 | 39.96^**^ | 38.90^**^ | - | - | $p_{1}=0.0236,\omega=169.9797$ $\beta(p=0.4493,q=0.7257)$ | 23, 2^*^, 1^**^ |
| DX8 | *Bd* | 0.8835 | 100.35^**^ | 100.02^**^ | 100.02^**^ | $p_{0}=0.4734,\omega_{0}=0.0000$ $p_{1}=0.3725,\omega_{1}=1.0000$ $p_{2}=0.1541,\omega_{2}=4.7513$ | 101, 18^*^, 8^**^ | $p_{1}=0.1660,\omega=4.5517$ $\beta(p=0.0126,q=0.0163)$ | 146, 40^*^, 16^**^ |
|  | oomycetes | 0.5229 | 86.52^**^ | 99.79^**^ | 82.72^**^ | $p_{0}=0.5227,\omega_{0}=0.0941$ $p_{1}=0.4020,\omega_{1}=1.0000$ $p_{2}=0.0754,\omega_{2}=6.0483$ | 41, 12^*^, 5^**^ | $p_{1}=0.0945,\omega=5.3047$ $\beta(p=0.2479,q=0.2952)$ | 59, 13^*^,6^**^ |
| DXX-DXV | *Bd* | 0.6522 | 23.36^**^ | 24.00^**^ | 23.10^**^ | $p_{0}=0.4260,\omega_{0}=0.0000$ $p_{1}=0.3876,\omega_{1}=1.0000$ $p_{2}=0.1864,\omega_{2}=2.9195$ | 114, 2^*^ | $p_{1}=0.2230,\omega=2.6785$ $\beta(p=0.0235,q=0.0304)$ | 164, 22^*^, 3^**^ |
|  | oomycetes | 0.5923 | 169.59^**^ | 207.20^**^ | 159.34^**^ | $p_{0}=0.5518,\omega_{0}=0.1289$ $p_{1}=0.3664,\omega_{1}=1.0000$ $p_{2}=0.0818,\omega_{2}=11.3186$ | 27, 16^*^, 12^**^ | $p_{1}=0.0944,\omega=9.8335$ $\beta(p=0.7937,q=1.0542)$ | 31, 17^*^, 14^**^ |
| DXX-DHA | *Bd* | 0.7843 | 99.09^**^ | 98.83^**^ | 98.65^**^ | $p_{0}=0.4690,\omega_{0}=0.0000$ $p_{1}=0.4050,\omega_{1}=1.0000$ $p_{2}=0.1260,\omega_{2}=4.9696$ | 61, 18^*^, 8^*^ | $p_{1}=0.1371,\omega=4.7019$ $\beta(p=0.0220,q=0.0279)$ | 91, 29^*^, 13^**^ |
|  | oomycetes | 0.6320 | 69.96^**^ | 71.46^**^ | 69.43^**^ | $p_{0}=0.4309,\omega_{0}=0.0247$ $p_{1}=0.5164,\omega_{1}=1.0000$ $p_{2}=0.0526,\omega_{2}=9.9395$ | 28, 11^*^, 4^**^ | $p_{1}=0.0546,\omega=9.6020$ $\beta(p=0.0202,q=0.0168)$ | 28, 11^*^, 4^**^ |
| DFA-DDC | *Bd* | 0.6880 | 58.05^**^ | 58.48^**^ | 58.03^**^ | $p_{0}=0.5433,\omega_{0}=0.0000$ $p_{1}=0.3756,\omega_{1}=1.0000$ $p_{2}=0.0811,\omega_{2}=17.5158$ | 36, 17^*^, 8^**^ | $p_{1}=0.0817,\omega=17.0427$ $\beta(p=0.0050,q=0.0075)$ | 42, 18^*^, 10^**^ |
|  | oomycetes | 0.2560 | 0 | 0.16 | 0 | - | - | - | - |
| DN17 | *Bd* | 0.4980 | 28.56^**^ | 28.82^**^ | 28.51^**^ | $p_{0}=0.7565,\omega_{0}=0.0000$ $p_{1}=0.1523,\omega_{1}=1.0000$ $p_{2}=0.0912,\omega_{2}=10.8747$ | 25, 8^*^, 3^**^ | $p_{1}=0.0935,\omega=10.7287$ $\beta(p=0.0153,q=0.0841)$ | 28, 9^*^, 5^**^ |
|  | oomycetes | 0.4726 | 82.51^**^ | 93.84^**^ | 87.67^**^ | $p_{0}=0.4550,\omega_{0}=0.0967$ $p_{1}=0.4547,\omega_{1}=1.0000$ $p_{2}=0.0903,\omega_{2}=8.2586$ | 29, 13^*^, 9^**^ | $p_{1}=0.1055,\omega=6.1185$ $\beta(p=0.5197,q=0.8570)$ | 40, 18^*^, 11^**^ |

1. The $d_{N}/d_{S}$ ratio average across all sites and lineages under PAML model M0.

2. Double asterisks represent $p<0.01$ for chi-square ($\chi^{2}$) test, while single asterisk represents $p<0.05$.

3. Positively selected sites, the number of amino acid residues with the posterior probability (PP) of positive selection greater than 0.05. Double asterisks represent the number of sites with PP >0.99, while single asterisk represents PP >0.95.

**Table S3. Results of positive selection using** branch-site models

| Gene family | Model | $\ln L$ | Parameter estimates | PSS |
| --- | --- | --- | --- | --- |
| serine peptidase | A_null_ | -24280.5596 | $p_{0}=\text{0.5906, }p_{1}=\text{0.3718, }p_{2a}=\text{0.0231, }p_{2b}=0.0145$ Background: $\omega_{0}=0.2782,\text{ }\omega_{1}=1.0000,\text{ }\omega_{2a}=0.2782,\text{ }\omega_{2b}=1.0000$ Foreground: $\omega_{0}=0.2782,\text{ }\omega_{1}=1.0000,\text{ }\omega_{2a}=1.0000,\text{ }\omega_{2b}=1.0000$ |  |
|  | A | -24278.1363^*^ | $p_{0}=\text{0.5171, }p_{1}=\text{0.3186, }p_{2a}=\text{0.1017, }p_{2b}=0.0626$ Background: $\omega_{0}=0.2770,\text{ }\omega_{1}=1.0000,\text{ }\omega_{2a}=0.2770,\text{ }\omega_{2b}=1.0000$ Foreground: $\omega_{0}=0.2770,\text{ }\omega_{1}=1.0000,\text{ }\omega_{2a}=197.6290,\text{ }\omega_{1}=197.6290$ | 13 |
| DXX-DAB | A_null_ | -6101.3305 | $p_{0}=\text{0.4227, }p_{1}=\text{0.3908, }p_{2a}=\text{0.0970, }p_{2b}=0.0896$ Background: $\omega_{0}=0.1003,\text{ }\omega_{1}=1.0000,\text{ }\omega_{2a}=0.1003,\text{ }\omega_{2b}=1.0000$ Foreground: $\omega_{0}=0.1003,\text{ }\omega_{1}=1.0000,\text{ }\omega_{2a}=1.0000,\text{ }\omega_{2b}=1.0000$ |  |
|  | A | -6101.3305 | $p_{0}=\text{0.4227, }p_{1}=\text{0.3908, }p_{2a}=\text{0.0970, }p_{2b}=0.0896$ Background: $\omega_{0}=0.1003,\text{ }\omega_{1}=1.0000,\text{ }\omega_{2a}=0.1003,\text{ }\omega_{2b}=1.0000$ Foreground: $\omega_{0}=0.1003,\text{ }\omega_{1}=1.0000,\text{ }\omega_{2a}=1.0000,\text{ }\omega_{1}=1.0000$ | - |
| DFB | A_null_ | -15292.8392 | $p_{0}=\text{0.3557, }p_{1}=\text{0.3887, }p_{2a}=\text{0.1221, }p_{2b}=0.1335$ Background: $\omega_{0}=0.1047,\text{ }\omega_{1}=1.0000,\text{ }\omega_{2a}=0.1047,\text{ }\omega_{2b}=1.0000$ Foreground: $\omega_{0}=0.1047,\text{ }\omega_{1}=1.0000,\text{ }\omega_{2a}=1.0000,\text{ }\omega_{2b}=1.0000$ |  |
|  | A | -15292.8394 | $p_{0}=\text{0.2862, }p_{1}=\text{0.3128, }p_{2a}=\text{0.1916, }p_{2b}=0.2094$ Background: $\omega_{0}=0.1047,\text{ }\omega_{1}=1.0000,\text{ }\omega_{2a}=0.1047,\text{ }\omega_{2b}=1.0000$ Foreground: $\omega_{0}=0.1003,\text{ }\omega_{1}=1.0000,\text{ }\omega_{2a}=2.6825,\text{ }\omega_{2b}=2.6825$ | - |
| DX8 | A_null_ | -11680.5755 | $p_{0}=\text{0.3544, }p_{1}=\text{0.3702, }p_{2a}=\text{0.1347, }p_{2b}=0.1407$ Background: $\omega_{0}=0.1073,\text{ }\omega_{1}=1.0000,\text{ }\omega_{2a}=0.1073,\text{ }\omega_{2b}=1.0000$ Foreground: $\omega_{0}=0.1073,\text{ }\omega_{1}=1.0000,\text{ }\omega_{2a}=1.0000,\text{ }\omega_{2b}=1.0000$ |  |
|  | A | -11675.7478^**^ | $p_{0}=\text{0.2882, }p_{1}=\text{0.3195, }p_{2a}=\text{0.1760, }p_{2b}=0.1663$ Background: $\omega_{0}=0.1228,\text{ }\omega_{1}=1.0000,\text{ }\omega_{2a}=0.1228,\text{ }\omega_{2b}=1.0000$ Foreground: $\omega_{0}=0.1228,\text{ }\omega_{1}=1.0000,\text{ }\omega_{2a}=28.8214,\text{ }\omega_{2b}=28.8214$ | 104 |
| DXX-DXV | A_null_ | -11622.7872 | $p_{0}=\text{0.5439, }p_{1}=\text{0.2926, }p_{2a}=\text{0.1063, }p_{2b}=0.0572$ Background: $\omega_{0}=0.1172,\text{ }\omega_{1}=1.0000,\text{ }\omega_{2a}=0.1172,\text{ }\omega_{2b}=1.0000$ Foreground: $\omega_{0}=0.1172,\text{ }\omega_{1}=1.0000,\text{ }\omega_{2a}=1.0000,\text{ }\omega_{2b}=1.0000$ |  |
|  | A | -11620.6559^*^ | $p_{0}=\text{0.5352, }p_{1}=\text{0.2818, }p_{2a}=\text{0.1199, }p_{2b}=0.0631$ Background: $\omega_{0}=0.1215,\text{ }\omega_{1}=1.0000,\text{ }\omega_{2a}=0.1215,\text{ }\omega_{2b}=1.0000$ Foreground: $\omega_{0}=0.1215,\text{ }\omega_{1}=1.0000,\text{ }\omega_{2a}=37.9350,\text{ }\omega_{2b}=37.9350$ | 31 |
| DXX-DHA | A_null_ | -8100.5876 | $p_{0}=\text{0.5211, }p_{1}=\text{0.3416, }p_{2a}=\text{0.0829, }p_{2b}=0.0544$ Background: $\omega_{0}=0.1358,\text{ }\omega_{1}=1.0000,\text{ }\omega_{2a}=0.1358,\text{ }\omega_{2b}=1.0000$ Foreground: $\omega_{0}=0.1358,\text{ }\omega_{1}=1.0000,\text{ }\omega_{2a}=1.0000,\text{ }\omega_{2b}=1.0000$ |  |
|  | A | -8100.5876 | $p_{0}=\text{0.5211, }p_{1}=\text{0.3416, }p_{2a}=\text{0.0829, }p_{2b}=0.0544$ Background: $\omega_{0}=0.1358,\text{ }\omega_{1}=1.0000,\text{ }\omega_{2a}=0.1358,\text{ }\omega_{2b}=1.0000$ Foreground: $\omega_{0}=0.1358,\text{ }\omega_{1}=1.0000,\text{ }\omega_{2a}=1.0000,\text{ }\omega_{2b}=1.0000$ | - |
| DFA-DDC | A_null_ | -4466.5119 | $p_{0}=\text{0.6701, }p_{1}=\text{0.2643, }p_{2a}=\text{0.0470, }p_{2b}=0.0185$ Background: $\omega_{0}=0.1406,\text{ }\omega_{1}=1.0000,\text{ }\omega_{2a}=0.1406,\text{ }\omega_{2b}=1.0000$ Foreground: $\omega_{0}=0.1406,\text{ }\omega_{1}=1.0000,\text{ }\omega_{2a}=1.0000,\text{ }\omega_{2b}=1.0000$ |  |
|  | A | -4464.0021^*^ | $p_{0}=\text{0.6521, }p_{1}=\text{0.2365, }p_{2a}=\text{0.0817, }p_{2b}=0.0296$ Background: $\omega_{0}=0.1457,\text{ }\omega_{1}=1.0000,\text{ }\omega_{2a}=0.1457,\text{ }\omega_{2b}=1.0000$ Foreground: $\omega_{0}=0.1457,\text{ }\omega_{1}=1.0000,\text{ }\omega_{2a}=72.8358,\text{ }\omega_{2b}=72.8358$ | 11 |
| DN17 | A_null_ | -4466.5119 | $p_{0}=\text{0.4989, }p_{1}=\text{0.2541, }p_{2a}=\text{0.1637, }p_{2b}=0.0834$ Background: $\omega_{0}=0.1619,\text{ }\omega_{1}=1.0000,\text{ }\omega_{2a}=0.1619,\text{ }\omega_{2b}=1.0000$ Foreground: $\omega_{0}=0.1619,\text{ }\omega_{1}=1.0000,\text{ }\omega_{2a}=1.0000,\text{ }\omega_{2b}=1.0000$ |  |
|  | A | -4464.0021^*^ | $p_{0}=\text{0.5000, }p_{1}=\text{0.2383, }p_{2a}=\text{0.1772, }p_{2b}=0.0845$ Background: $\omega_{0}=0.1690,\text{ }\omega_{1}=1.0000,\text{ }\omega_{2a}=0.1690,\text{ }\omega_{2b}=1.0000$ Foreground: $\omega_{0}=0.1690,\text{ }\omega_{1}=1.0000,\text{ }\omega_{2a}=9.4812,\text{ }\omega_{2b}=9.4812$ | 35 |


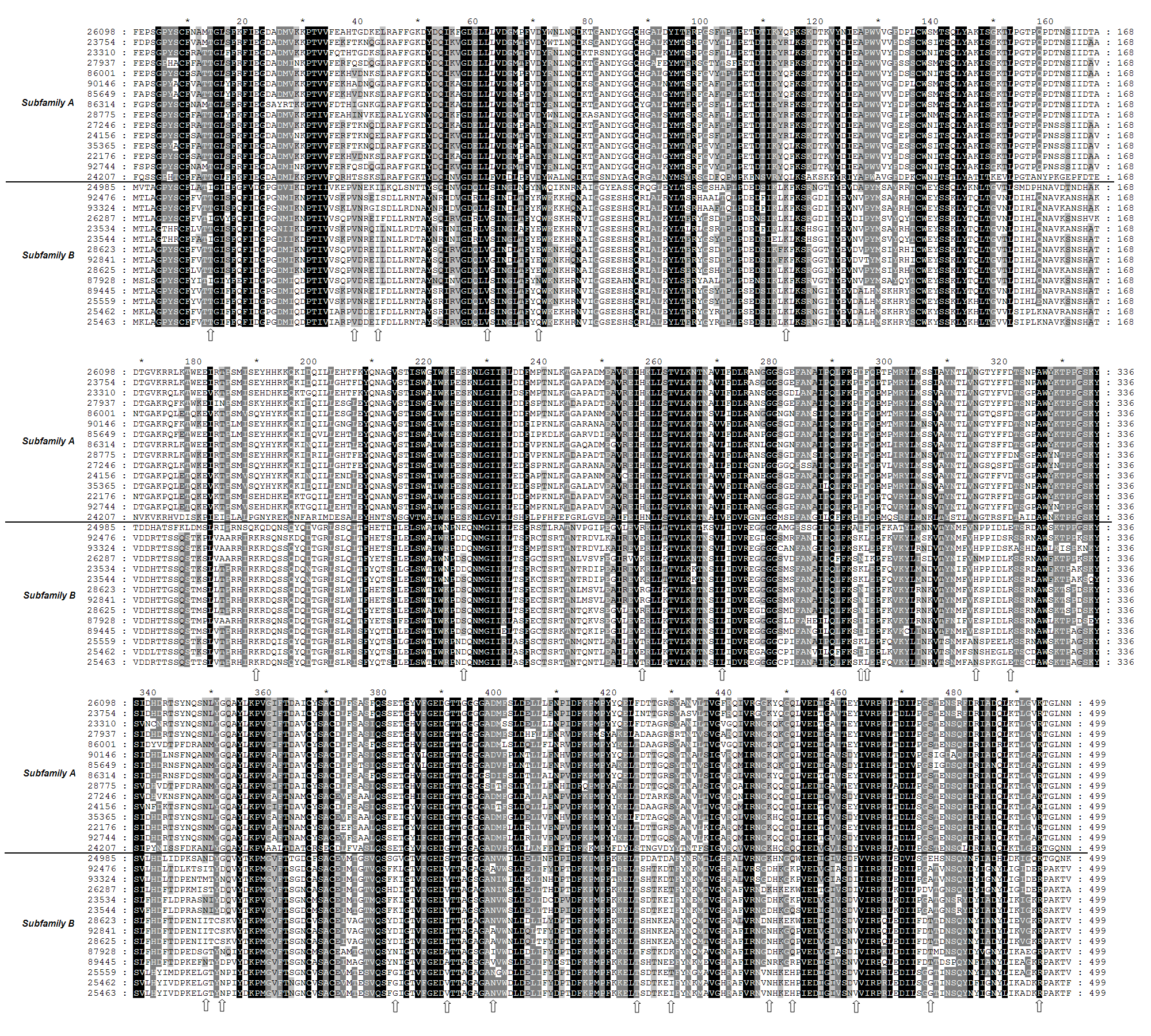


**Fig. S2.** Amino acid residues that are critical to functional divergence between serine peptidase clades A and B. Gaps of the alignment have been deleted using PAL2NAL. The critical amino acid residues for functional divergence with posterior probability higher than 0.95 are illustrated by the arrows.
