# Supplementary material for: Evidence for acquisition of virulence effectors in pathogenic chytrids
Source: BMC Evol Biol. 2011 Jul 8;11:195. doi: 10.1186/1471-2148-11-195 (PMC3161006; doi:10.1186/1471-2148-11-195)
Supplement: Additional file 4 — Data sources for Bd isolates and oomycetes used in the analyses. This file shows data types and download sites for two Bd isolates and seven oomycetes used in the analyses. [file 1471-2148-11-195-S4.PDF]

Table S4. Data sources for the two chytrid isolates and seven oomycetes used in the analyses

Groups	Species names	Database types	Download sites
chytrids	*Batrachochytrium dendrobatidis* JAM81	genome	http://genome.jgi-psf.org/Batde5/Batde5.download.ftp.html
	Batrachochytrium dendrobatidis JEL423	genome	http://www.broadinstitute.org/annotation/genome/batrachochytrium_dendrobatidis/MultiDownloads.html
oomycetes	*Phytophthora infestans*	genome	http://www.broadinstitute.org/annotation/genome/phytophthora_infestans/MultiDownloads.html
	Phytophthora sojae	genome	http://genome.jgi-psf.org/Physo1_1/Physo1_1.download.ftp.html
	Phytophthora capsici	genome	http://genome.jgi-psf.org/PhycaF7/PhycaF7.download.ftp.html
	Phytophthora ramorum	genome	http://genome.jgi-psf.org/Phyra1_1/Phyra1_1.download.ftp.html
	Pythium ultimum	genome	http://pythium.plantbiology.msu.edu/download.html
	Phytophthora brassicae	ESTs	http://www.ncbi.nlm.nih.gov/nucest
	Phytophthora parasitica	ESTs	http://www.ncbi.nlm.nih.gov/nucest
	Aphanomyces euteiches	ESTs	http://www.polebio.scsv.ups-tlse.fr/aphano/
