# Supplementary material for: Evidence for acquisition of virulence effectors in pathogenic chytrids
Source: BMC Evol Biol. 2011 Jul 8;11:195. doi: 10.1186/1471-2148-11-195 (PMC3161006; doi:10.1186/1471-2148-11-195)
Supplement: Additional file 5 — Two different conserved N-terminal domains of Bd CRN proteins. This file shows two different types of N-terminal domain for Bd CRN proteins. These domain types are also indicated in Table 1. [file 1471-2148-11-195-S5.PDF]

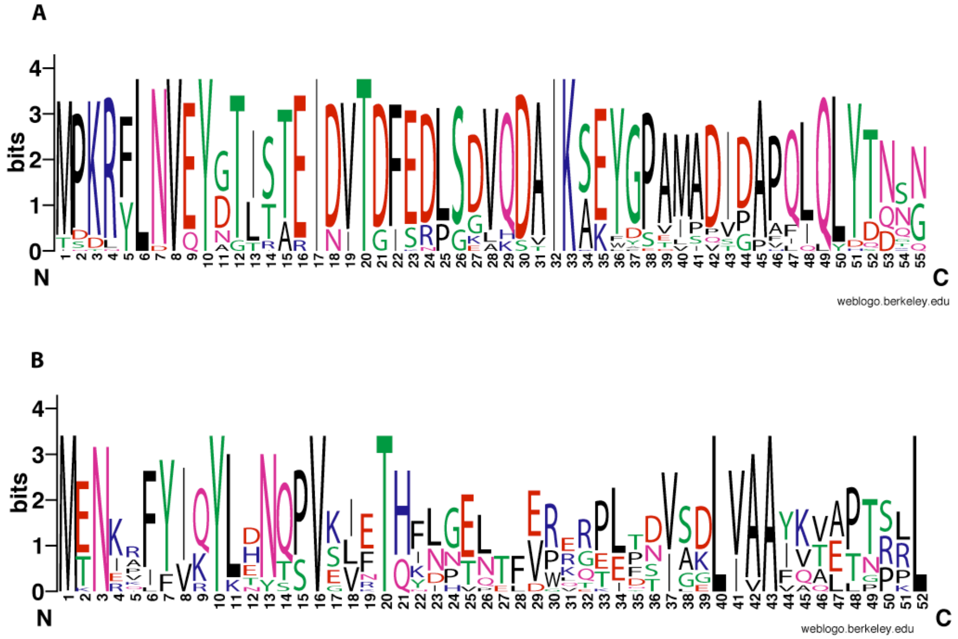

Fig. S3. Two different conserved N-terminal domains of *Bd* CRN proteins. Profile A was generated from an alignment of the first 55 amino acids of 42 DHA subfamily proteins, 5 DAB subfamily proteins, and 3 DXV subfamily proteins. Profile B was generated from an alignment of the first 52 amino acids of 3 DDC subfamily proteins, 10 DFB subfamily proteins, 6 DX8 subfamily proteins, and 2 DXV subfamily proteins. The two conserved motifs in profile B are "FYIQYL(D/H/E/T)NQPV" (6-11aa) and "LVAA"(40-43aa). These logos were generated using WebLogo (http://weblogo.berkeley.edu).
