# Supplementary material for: Evidence for acquisition of virulence effectors in pathogenic chytrids
Source: BMC Evol Biol. 2011 Jul 8;11:195. doi: 10.1186/1471-2148-11-195 (PMC3161006; doi:10.1186/1471-2148-11-195)
Supplement: Additional file 2 — LFLAK domain of the CRN protein family in oomycetes and Bd. This file shows that the LFLAX domain of the CRN protein family is largely conserved between oomycetes and Bd. [file 1471-2148-11-195-S2.PDF]

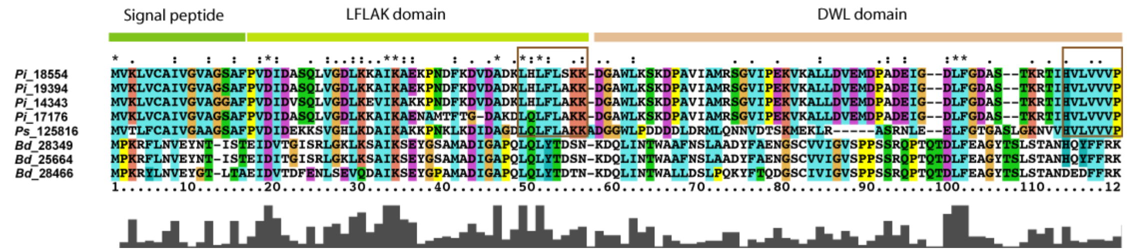

Fig. S1. LFLAK domain of the CRN protein family in oomycetes is conserved in some *Bd* homologs. Locations of signal peptide, LFLAK domain, and DWL domain in oomycetes are indicated using colored bars. The conserved LQLFLAK motif and HVLVVVP motif in oomycete sequences are indicated in boxes.
